# Food insecurity and subjective cognitive complaints among adults aged ≥ 65 years from low- and middle-income countries

**DOI:** 10.1007/s00394-023-03226-5

**Published:** 2023-08-07

**Authors:** Lee Smith, Guillermo F. López Sánchez, Jae Il Shin, Karel Kostev, Benjamin R. Underwood, Hans Oh, Pinar Soysal, Nicola Veronese, Felipe Schuch, Mark A. Tully, Ai Koyanagi

**Affiliations:** 1 Centre for Health Performance and Wellbeing, Anglia Ruskin University, Cambridge, UK; 2 Division of Preventive Medicine and Public Health, Department of Public Health Sciences, School of Medicine, University of Murcia, Murcia, Spain; 3 Department of Pediatrics, Yonsei University College of Medicine, Seoul, South Korea; 4 Severance Underwood Meta-research Center, Institute of Convergence Science, Yonsei University, Seoul, South Korea; 5 University Clinic of Marburg, Marburg, Germany; 6 Cambridgeshire and Peterborough NHS Foundation Trust and Department of Psychiatry, University of Cambridge, Cambridge, UK; 7 Suzanne Dworak Peck School of Social Work, University of Southern California, Los Angeles, CA USA; 8 Department of Geriatric Medicine, Faculty of Medicine, Bezmialem Vakif University, Istanbul, Turkey; 9 Department of Internal Medicine, Geriatrics Section, University of Palermo, Palermo, Italy; 10 Department of Sports Methods and Techniques, Federal University of Santa Maria, Santa Maria, Brazil; 11 Faculty of Health Sciences, Universidad Autónoma de Chile, Providencia, Chile; 12 School of Medicine, Ulster University, Londonderry, Northern Ireland, UK; 13 Research and Development Unit, Parc Sanitari Sant Joan de Déu, CIBERSAM, ISCIII, Dr. Antoni Pujadas, Sant Boi de Llobregat, Barcelona, Spain; 14 ICREA, Pg. Lluis Companys 23, 08010 Barcelona, Spain

**Keywords:** Food insecurity, Subjective cognitive complaints, Low- and middle-income countries, Older adults

## Abstract

**Purpose:**

To date, no study has investigated the association between food insecurity and subjective cognitive complaints (SCC). Thus, the aims of the present study were to examine this association among older adults in low- and middle-income countries (LMICs), and to identify the potential mediators in this association, given the importance of SCC in dementia risk among older people, and the projected particularly large increase in dementia in this setting.

**Methods:**

Cross-sectional, community-based, nationally representative data from the World Health Organization (WHO) Study on global AGEing and Adult Health (SAGE) collected between 2007 and 2010 were analyzed. Two questions on subjective memory and learning complaints in the past 30 days were used to create a SCC scale ranging from 0 (No SCC) to 100 (worse SCC). Past 12 month food insecurity was assessed with two questions on frequency of eating less and hunger due to lack of food. Multivariable linear regression and mediation (Karlson–Holm–Breen method) analyses were conducted to assess associations.

**Results:**

Data on 14,585 individuals aged ≥ 65 years [mean (SD) age 72.6 (11.5) years; 55.0% females] were analyzed. Severe food insecurity (vs. no food insecurity) was associated with 9.16 (95% CI = 6.95–11.37) points higher mean SCC score. Sleep/energy (mediated% 37.9%; P < 0.001), perceived stress (37.2%; P = 0.001), and depression (13.7%; P = 0.008) partially explained the association between severe food insecurity and SCC.

**Conclusion:**

Food insecurity was associated with SCC among older adults in LMICs. Future studies should assess whether addressing food insecurity among older adults in LMICs can improve cognitive health.

**Supplementary Information:**

The online version contains supplementary material available at 10.1007/s00394-023-03226-5.

## Introduction

Dementia is a syndrome that leads to progressive deterioration in memory and other cognitive domains (i.e., the ability to process thought) beyond what might be expected from the usual consequences of biological ageing, and which impairs functional ability [1]. Currently more than 55 million people live with dementia worldwide, and there are nearly 10 million new cases every year. Dementia is currently the seventh leading cause of death among all diseases and one of the major causes of disability and dependency among older people globally [1]. In 2010, 58% of all people with dementia were residing in low- and middle-income countries (LMICs), with this proportion anticipated to rise to 63% in 2030 and 71% in 2050 [2]. Given the high and increasing prevalence of dementia, and the fact that there is currently no disease modifying treatment for the underlying causes of dementia, identifying modifiable risk factors for dementia or its precursory stage may be of prime importance to prevent or delay the onset of dementia.

Subjective cognitive complaints (SCC) refer to concerns regarding cognition of people both with and without objective evidence of memory impairment [3], and have been found to be a predictor of the future development of dementia among older people, even in the absence of objective cognitive impairment [4]. In a recent meta-analysis including 46 studies with more than 74,000 participants, subjective cognitive decline was associated with an approximately twofold increased risk of developing dementia or mild cognitive impairment [5]. Moreover, biological changes associated with an increased risk of dementia, such as increases in white matter lesions, temporal atrophy, and altered cerebrospinal fluid biomarkers have also been observed in individuals with SCC [6–8]. SCC may also serve as an early clinical marker of dementia and help predict subtle cognitive changes at earlier stages than objective measures [4].

Currently, there is increasing interest in the role of food insecurity on cognitive decline [9]. Food insecurity is defined as “limited or uncertain availability of nutritionally adequate and safe foods or limited or uncertain ability to acquire food in socially acceptable ways”, and is particularly highly prevalent in LMICs [10–12]. Several previous studies from high-income countries and LMICs have shown that food insecurity may be associated with cognitive decline. For example, one longitudinal study from the US found that food insecurity at baseline was associated with a decline in global cognitive function over the following two years [13]. Furthermore, in a cross-sectional study conducted in India, it was observed that respondents from food secure households were 14% less likely to have word recall problems [14]. In addition, another cross-sectional study from South Africa observed a positive association between food insecurity and mild cognitive impairment [15]. However, to the best of the authors’ knowledge, there are no existing studies specifically on the association between food insecurity and SCC.

It is possible for food insecurity to lead to SCC via several mechanisms. For example, malnutrition and lower vitamin B1 and B12 levels, which are common in food insecurity [16], are independently associated with greater risk of white matter hyperintensities [17], which may increase risk of cognitive impairment and dementia [18]. Cognitive dysfunction has been well described in anorexia nervosa, and several studies have found improvement with treatment, suggesting a direct correlation with malnutrition and cognitive function [19]. Furthermore, the psychological consequences of food insecurity (e.g., sleep problems, perceived stress, anxiety, depression) may also lead to SCC. For example, food insecurity may increase levels of perceived stress owing to “worry” in relation to accessing food over time. In turn, perceived stress may increase risk for SCC by impairing working memory.

Given this background, the aim of the present study was to examine the association between food insecurity and SCC in a large nationally representative sample of adults aged ≥ 65 years from six LMICs, an association which to the best of our knowledge has not been previously described. A further aim was to examine to what extent anxiety, depression, perceived stress, and sleep problems mediate the food insecurity-SCC relationship.

## Methods

The statistical analysis was undertaken using Stata 14.2 (Stata Corp LP, College station, Texas). Data from the Study on Global AGEing and Adult Health (SAGE) were analysed. This survey was conducted in China, Ghana, India, Mexico, Russia, and South Africa between 2007 and 2010. Based on the World Bank classification at the time of the survey, Ghana was the only low-income country, and China and India were lower middle-income countries at the time of data collection, although China became an upper middle-income country in 2010. The remaining countries were upper middle-income countries. Details of the survey methodology have been published elsewhere [20]. Briefly, in order to obtain nationally representative samples, a multistage clustered sampling design method was used. The sample consisted of adults aged ≥ 18 years with oversampling of those aged ≥ 50 years. The present analysis was restricted to those aged ≥ 65 years. Trained interviewers conducted face-to-face interviews using a standard questionnaire. Standard translation procedures were undertaken to ensure comparability between countries. If a respondent was unable to undertake the interview because of limited cognitive function, then a separate questionnaire was administered to a proxy respondent. These individuals were not included in the current study. The survey response rates were: China 93%; Ghana 81%; India 68%; Mexico 53%; Russia 83%; and South Africa 75%. Sampling weights were constructed to adjust for the population structure at the time of the survey as reported by the United Nations Statistical Division. Ethical approval was obtained from the WHO Ethical Review Committee and local ethics research review boards (no ethical approval number). Written informed consent was obtained from all participants.

### Subjective cognitive complaints (SCC)

SCC were assessed with two questions: (a) “Overall in the last 30 days, how much difficulty did you have with concentrating or remembering things?”; and (b) “Overall in the last 30 days, how much difficulty did you have in learning a new task (for example, learning how to get to a new place, learning a new game, learning a new recipe etc.)?” Each item was scored on a five-point scale: none (score = 1), mild (score = 2), moderate (score = 3), severe (score = 4), and extreme/cannot do (score = 5). Since these answer options were an ordered categorical scale, we conducted factor analysis with polychoric correlations to incorporate the covariance structure of the answers provided for individual questions measuring a similar construct [21, 22]. The principal component method was used for factor extraction, while factor scores were obtained using the regression scoring method. These factor scores were later converted to scores ranging from 0 to 100 to create a SCC scale with higher values representing worse subjective cognitive function [23]. The results of the factor analysis are provided in Table S2 of the Appendix.

### Food insecurity

Food insecurity was defined by the two following questions: “In the last 12 months, how often did you ever eat less than you felt you should because there wasn’t enough food?” and “In the last 12 months, were you ever hungry, but didn’t eat because you couldn’t afford enough food?” Both of these questions had as response options: every month (coded = 1); almost every month (coded = 2); some months, but not every month (coded = 3); only in 1 or 2 months (coded = 4); never (coded = 5). These items were based on similar items found in food security questionnaires such as the US Household Food Security Survey Module and National Health and Nutrition Examination Survey (NHANES) Food Security module. As in a previous SAGE study, those who answered 1 through 3 to both questions or answered 1 to either item were categorized as severely food insecure. Those who did not fulfill the criteria for severe food insecurity, but answered 2 through 4 for either question, were coded as moderately food insecure. Those who answered 5 to both items were categorized as food secure [24]. We also used the dichotomous variable of severe food insecurity (i.e., severe or none/moderate) in some analyses.

### Mediators

The potential mediators (i.e., anxiety, depression, perceived stress, sleep/energy) were chosen based on the possibility that they can be the consequence of food insecurity, and the fact that they have previously been associated with cognitive decline [25–28]. Questions based on the World Mental Health Survey version of the Composite International Diagnostic Interview were used for the endorsement of past 12 month DSM-IV depression [29]. Anxiety symptoms was assessed by the question ‘Overall in the past 30 days, how much of a problem did you have with worry or anxiety’ with response alternatives: ‘none’, ‘mild’, ‘moderate’, ‘severe’, and ‘extreme’. In accordance with previous publications, those who answered ‘severe’ and ‘extreme’ were considered to have anxiety [30, 31]. Perceived stress and sleep/energy were assessed with two questions each. The questions on perceived stress were taken from the Perceived Stress Scale [32]. The actual questions can be found in supplementary Table S1. Each item was scored on a five-point scale ranging from ‘none’ to ‘extreme/cannot do’ (sleep/energy) or from ‘never’ to ‘very often’ (perceived stress). For each of the two conditions, we used factor analysis with polychoric correlations to obtain a factor score which was later converted to scores ranging from 0 to 100 with higher values representing worse status [33].

### Control variables

The selection of the control variables included in this study was based on past literature [13], and included age (years), sex, years of education received, wealth quintiles, physical activity, smoking, alcohol use in the past 30 days, body mass index (BMI) based on measured weight and height (< 18.5, 18.5–24.9, 25.0–29.9, 30 kg/m^2^), and chronic physical conditions (diabetes, hypertension, stroke). A hierarchical ordered probit model was utilized to create an index of household asset ownership of durable goods, dwelling characteristics, and access to services (e.g., cooking fuel, sanitation, improved water), and based on this index, country-wise wealth quintiles were generated. Levels of physical activity were assessed with the Global Physical Activity Questionnaire and were classified as low, moderate, and high based on conventional cut-offs [34]. For smoking, the participant was first asked whether he/she had ever smoked tobacco or used smokeless tobacco. If the participant answered “No”, then he/she was considered to have never smoked. In case of an affirmative answer, the participant was prompted to the next question which asked about whether the participant currently uses tobacco products. Participants were divided into never, past, and current smokers based on these two questions. Diabetes and stroke were based solely on lifetime self-reported diagnosis. Hypertension was defined as having at least one of: systolic blood pressure ≥ 140 mmHg; diastolic blood pressure ≥ 90 mmHg; or self-reported diagnosis.

### Statistical analysis

The difference in sample characteristics was tested using Chi-squared tests and Student’s* t*-tests for categorical and continuous variables, respectively. Country-wise multivariable linear regression analysis [35] was conducted to assess the association between food insecurity (i.e., no, moderate, severe food insecurity) (exposure) and SCC (outcome) with no food insecurity as the reference category. To quantify the degree of between-country heterogeneity, the Higgin’s *I*^*2*^ was also calculated, and this represents the degree of heterogeneity that is not explained by sampling error with a value of < 40% often considered as negligible and 40–60% as moderate heterogeneity [36]. An overall estimate was obtained based on country-wise estimates by meta-analysis. Random effects meta-analysis was performed when the level of between-country heterogeneity was at least moderate. Otherwise, fixed effects meta-analysis was conducted.

Next, mediation analysis was conducted to gain an understanding of the extent to which anxiety, depression, perceived stress, and sleep/energy may explain the association between severe food insecurity and SCC using the overall sample. We used a dichotomous variable for food insecurity (i.e., severe food insecurity or not) as preliminary analysis showed that severe food insecurity is particularly strongly associated with SCC in the overall sample. We used the *khb* (Karlson–Holm–Breen) command in Stata [37] for the mediation analysis. This method decomposes the total effect (i.e., unadjusted for the mediator) of a variable into direct and indirect effects. Using this method, the percentage of the main association explained by the mediator can also be calculated (mediated percentage). Each potential mediator was included in the model individually, with the exception of the analysis where all mediators were included simultaneously in the model.

All regression analyses including the mediation analysis were adjusted for age, sex, education, wealth, physical activity, smoking, alcohol use, BMI, diabetes, hypertension, and stroke. The mediation analysis was additionally adjusted for country, and this was done by including dummy variables for each country in the model as in previous SAGE publications [38, 39]. The sample weighting and the complex study design were taken into account in all analyses. Results from the regression analyses are presented as b-coefficients with 95% confidence intervals (CIs). The level of statistical significance was set at P < 0.05. The statistical analysis was conducted in November 2021.

## Results

**Fig. 1:**
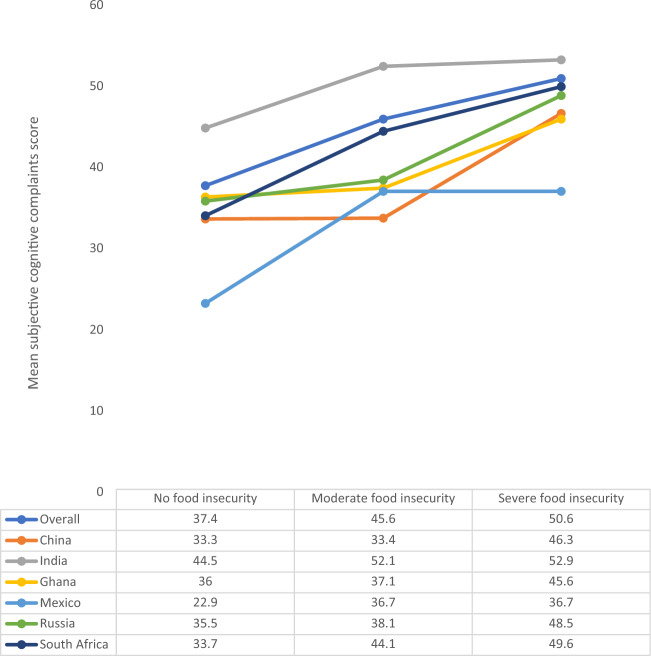
Mean subjective cognitive complaints score by level of food insecurity. The subjective cognitive complaints score ranged from 0 to 100 with higher scores representing worse subjective cognitive function

**Fig. 2:**
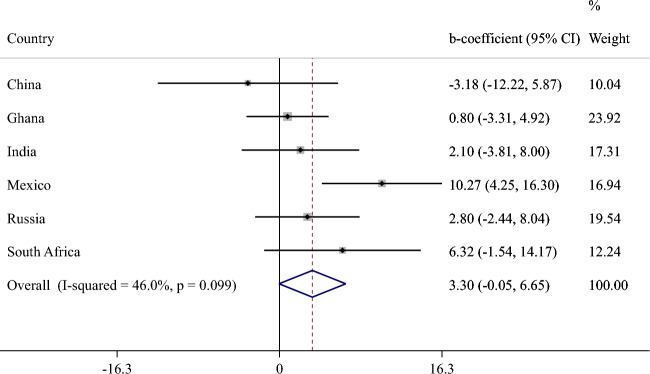
Association between moderate food insecurity (vs. no food insecurity) and subjective cognitive complaints (outcome) estimated by multivariable linear regression. CI Confidence interval. The subjective cognitive complaints score ranged from 0 to 100 with higher scores representing worse subjective cognitive function. Models are adjusted for age, sex, education, wealth, physical activity, smoking, alcohol use, body mass index, diabetes, hypertension, and stroke. Overall estimate was based on meta-analysis with random effects

**Fig. 3:**
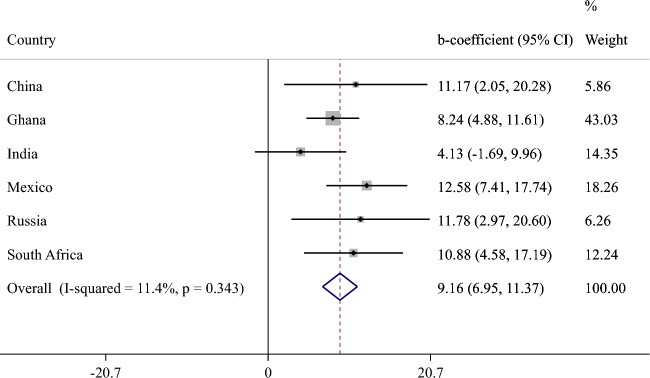
Association between severe food insecurity (vs. no food insecurity) and subjective cognitive complaints (outcome) estimated by multivariable linear regression. CI Confidence interval. The subjective cognitive complaints score ranged from 0 to 100 with higher scores representing worse subjective cognitive function. Models are adjusted for age, sex, education, wealth, physical activity, smoking, alcohol use, body mass index, diabetes, hypertension, and stroke. Overall estimate was based on meta-analysis with fixed effects

The final sample included 14,585 individuals aged ≥ 65 years [mean (SD) age 72.6 (11.5) years; 55.0% females]. The sample sizes by country were: China n = 5360; Ghana n = 1975; India n = 2441; Mexico n = 1375; Russia n = 1950; South Africa n = 1484. The overall prevalence of moderate and severe food insecurity was 6.7% and 5.0%, respectively. The country-wise prevalence of food insecurity is provided in Fig. S1 (Appendix). The prevalence of moderate food insecurity ranged from 0.8 (China) to 22.6% (Ghana), and that of severe food insecurity ranged from 0.3 (China) to 22.4% (Ghana). The sample characteristics are provided in Table 1. Compared to those without severe food insecurity, the prevalence of lower levels of wealth (P < 0.001), higher physical activity (P = 0.004), underweight (P < 0.001), anxiety (P < 0.001), and depression (P < 0.001) were higher among those with severe food insecurity, and levels of years of education lower (P < 0.001), and perceived stress and sleep/energy problems higher (P < 0.001). The sample characteristics by country are provided in Table S3 of the Appendix. Russia had a particularly high proportion of females and people with higher levels of education. The mean SCC sore (0–100) was higher in severe food insecurity compared to no food insecurity in all countries (Fig. 1). Meta-analysis with random effects based on country-wise estimates showed that moderate food insecurity (vs. no food insecurity) is not significantly associated with SCC scores (beta = 3.30; 95% CI = − 0.05, 6.65) with a moderate level of between-country heterogeneity (*I*^2^ = 46.0%) (Fig. 2). In order to identify the source of between-country heterogeneity, we deleted one country at a time and assessed the change in the *I*^2^. This analysis showed that a moderate level of between-country heterogeneity was maintained for all countries, with the exception of Mexico. Specifically, when Mexico was deleted from the analysis, the *I*^2^ was reduced to 0.0%. The estimates for severe food insecurity are shown in Fig. 3. Based on a meta-analysis with fixed effects, severe food insecurity (vs. no food insecurity) was associated with a significant 9.16 (95% CI = 6.95–11.37) points higher mean SCC score, with a low level of between-country heterogeneity (*I*^2^ = 11.4%). Sleep/energy (mediated% 37.9%; P < 0.001), perceived stress (37.2%; P < 0.001), and depression (13.7%; P = 0.008) were found to be significant mediators in the association between severe food insecurity and SCC, but anxiety was not (Table 2). All potential mediators collectively explained 67.1% of the association.

**Table 1:** Sample characteristics (overall and by severe food insecurity)

			Severe food insecurity		
Characteristic		Overall	No	Yes	P value^a^
Age (years)	Mean (SD)	72.6 (11.5)	72.5 (11.1)	72.5 (14.9)	0.967
Sex	Female	55.0 (0.9)	54.5 (0.9)	62.2 (3.7)	0.053
	Male	45.0 (0.9)	45.5 (0.9)	37.8 (3.7)	
Education (years)	Mean (SD)	5.2 (9.3)	5.3 (9.2)	3.6 (10.7)	< 0.001
Wealth	Poorest	21.7 (1.1)	20.6 (1.1)	41.6 (4.2)	< 0.001
	Poorer	21.0 (1.1)	20.6 (1.1)	26.9 (4.1)	
	Middle	20.4 (0.9)	20.5 (0.9)	18.6 (3.2)	
	Richer	17.5 (0.8)	18.0 (0.8)	7.6 (1.5)	
	Richest	19.4 (1.1)	20.2 (1.1)	5.3 (1.1)	
Physical activity	High	35.2 (1.1)	34.8 (1.2)	43.7 (4.1)	0.004
	Moderate	25.2 (0.8)	25.7 (0.8)	15.1 (2.2)	
	Low	39.6 (1.0)	39.5 (1.1)	41.2 (3.5)	
Smoking	Never	62.2 (1.2)	62.5 (1.2)	56.8 (3.9)	0.129
	Current	29.3 (1.2)	29.0 (1.2)	35.7 (4.0)	
	Past	8.5 (0.4)	8.5 (0.4)	7.5 (1.5)	
Alcohol consumption	No	86.1 (0.7)	86.0 (0.7)	87.1 (2.4)	0.673
	Yes	13.9 (0.7)	14.0 (0.7)	12.9 (2.4)	
Body mass index	< 18.5	19.3 (1.1)	18.5 (1.1)	34.8 (3.8)	< 0.001
(kg/m^2^)	18.5–24.9	46.4 (1.1)	46.9 (1.2)	35.5 (3.7)	
	25.0–29.9	23.9 (1.0)	24.4 (1.0)	14.9 (3.9)	
	≥ 30.0	10.4 (0.7)	10.2 (0.8)	14.8 (2.8)	
Diabetes	No	91.4 (0.5)	91.3 (0.6)	93.0 (2.3)	0.521
	Yes	8.6 (0.5)	8.7 (0.6)	7.0 (2.3)	
Stroke	No	95.4 (0.3)	95.3 (0.3)	96.4 (1.2)	0.423
	Yes	4.6 (0.3)	4.7 (0.3)	3.6 (1.2)	
Hypertension	No	36.6 (1.2)	36.5 (1.2)	40.7 (4.0)	0.239
	Yes	63.4 (1.2)	63.5 (1.2)	59.3 (4.0)	
Anxiety	No	90.3 (0.7)	90.9 (0.7)	78.6 (3.0)	< 0.001
	Yes	9.7 (0.7)	9.1 (0.7)	21.4 (3.0)	
Depression	No	93.5 (0.5)	94.2 (0.5)	81.1 (2.6)	< 0.001
	Yes	6.5 (0.5)	5.8 (0.5)	18.9 (2.6)	
Perceived stress^b^	Mean (SD)	43.3 (44.9)	42.5 (43.7)	57.6 (56.5)	< 0.001
Sleep/energy^b^	Mean (SD)	33.1 (49.7)	32.3 (48.7)	47.6 (57.7)	< 0.001

Data are % (standard error) unless otherwise stated

*SD* standard deviation

^a^P value was based on Chi-squared test and Student’s *t* test for categorical and continuous variables, respectively

^b^Perceived stress and sleep/energy were based on a scale that ranged from 0 to 100 with higher scores representing worse conditions

**Table 2:** Mediators in the association between severe food insecurity and subjective cognitive complaints

Mediator	Effect	b-coefficient [95% CI]	P value	%Mediated^a^
Anxiety	Total	6.13 [2.45, 9.81]	0.001	NA
	Direct	5.20 [1.53, 8.87]	0.006	
	Indirect	0.93 [− 0.01, 1.88]	0.053	
Depression	Total	6.14 [2.36, 9.91]	0.001	13.7
	Direct	5.30 [1.51, 9.08]	0.006	
	Indirect	0.84 [0.22, 1.47]	0.008	
Perceived stress	Total	6.06 [2.39, 9.73]	0.001	37.2
	Direct	3.81 [0.15, 7.47]	0.042	
	Indirect	2.25 [1.19, 3.31]	< 0.001	
Sleep/energy	Total	6.19 [2.93, 9.46]	< 0.001	37.9
	Direct	3.85 [0.57, 7.12]	0.021	
	Indirect	2.35 [1.12, 3.57]	< 0.001	
All mediators	Total	6.12 [2.90, 9.34]	< 0.001	67.1
	Direct	2.01 [− 1.20, 5.22]	0.219	
	Indirect	4.10 [2.40, 5.80]	< 0.001	

The subjective cognitive complaints score ranged from 0 to 100 with higher scores representing worse subjective cognitive function

Models are adjusted for age, sex, education, wealth, physical activity, smoking, alcohol use, body mass index, diabetes, hypertension, stroke, and country

*CI* confidence interval

^a^Mediated percentage was only calculated in the presence of a significant indirect effect (P < 0.05)

## Discussion

### Main findings

In this large sample of adults aged ≥ 65 years from six LMICs, food insecurity was highly prevalent in most countries, and severe food insecurity (vs. no food insecurity) was significantly associated with higher mean SCC scores. Moreover, sleep/energy and perceived stress explained nearly 40% of the association between severe food insecurity and SCC, while depression explained 13.7%. Anxiety was not a significant mediator. Collectively, all the potential mediators explained 67.1% of the association between severe food insecurity and SCC. To the best of our knowledge, this is the first study on food insecurity and SCC.

### Interpretation of the findings

The results of our study concur with most of the previous studies on food insecurity and cognitive decline which have used objective cognitive measures or mild cognitive impairment as the outcome [9]. There are several plausible pathways that explain the food insecurity-SCC relationship. First, sleep/energy, perceived stress, and depression were all found to be significant mediators in the association. Hunger (one component of food insecurity) has been suggested to directly influence sleep quality, continuity and duration [27], and sleep problems in turn, can derange metabolic and endocrine function which can result in cognitive dysfunction, and thus SCC [40]. Next, food insecurity may increase levels of perceived stress by creating uncertain situations over the ability to sustain or access food over time. Furthermore, food insecurity may also increase the socioeconomic disparity at the household level and communities, which can affect overall psychological wellbeing [41]. Perceived stress may increase risk for SCC by impairing working memory and cognitive flexibility [42]. Specifically, stress may exert its negative effects on cognition through several physiological pathways pertaining to the central nervous, neuroendocrine, and immune systems. In terms of the neuroendocrine pathway, prolonged elevation of cortisol, which is a HPA axis response to chronic stress, may increase risk for stress-related cognitive decline [43]. Finally, food insecurity may increase risk for depression owing to poor nutrition or increased feelings of shame [25, 44, 45]. In turn, depression may increase risk for SCC via changes in brain structure and function, including in the prefrontal cortex, hippocampus, and amygdala. These regions are all involved in cognition, executive function (such as planning, decision-making, and reasoning), and emotion processing [46].

In our study, we found that about 2/3 of the association between severe food insecurity and SCC were explained by psychological factors assessed. As for the remainder, this is likely to be explained by factors which were not assessed in our study (e.g., suboptimal nutrition). For example, food insecurity is strongly associated with poor diets (e.g., high-fat diet, low vitamin and mineral consumption) [47]. A high-fat diet has been demonstrated to impair hippocampus-dependent memory function [48]. Furthermore, as previously discussed, lower vitamin B1 and B12 levels are independently associated with greater risk of white matter hyperintensities [17], which are associated with increased risk for cognitive impairment and dementia [18].

Interestingly, only Mexico demonstrated a significant positive association between moderate food insecurity and SCC. The reason why this may be is elusive but could be due to variations in the availability of healthy foods between countries. For example, while speculative, people exposed to moderate food insecurity in Mexico may be more likely to consume unhealthy food, which is detrimental to cognitive health [49, 50]. However, further research of a qualitative nature is needed to elucidate on the finding regarding Mexico.

### Implication of the study findings

Food insecurity has been reported to be associated with negative health outcomes such as cardiovascular disease and all-cause mortality [51]. Findings from the present study putatively support, pending further longitudinal and/or experimental research, the idea that interventions to address food insecurity among older people in LMICs may have the additional benefit of improving cognitive health and possibly decreasing the incidence of dementia, and that this effect may at least partly be due to improvements in sleep problems, perceived stress, and depression, which have all previously been reported to be risk factors for dementia [52–54]. Interventions to address food insecurity in LMICs may include the implementation of food banks which have been highly successful in high-income countries to tackle food insecurity [55]. Another successful intervention in high-income countries specifically targeted at older adults is home delivery of meals supported by governments [56, 57]. However, such initiatives are rare in LMICs and will require strong governmental “buy in” to implement. In LMICs, food insecurity affects predominantly rural areas. Therefore, increasing agricultural outputs could potentially be an effective means of alleviating food insecurity in the region. Policies supporting small-holder farmers may boost agricultural productivity, and as a consequence, income from agriculture, which constitutes rural households’ main source of revenue [58].

### Strengths and limitations

The use of large nationally representative datasets from LMICs, and the identification of potential mediators in the association between food insecurity and SCC are the strengths of the present study. However, the study results should be interpreted in light of their limitations. First, the study was cross-sectional in nature, and thus, temporal associations or causality could not be established. Relatedly, the potential mediators in our study were selected based on their possibility to be the consequence of food insecurity and a cause of SCC, but mediation and confounding are identical statistically, and thus, it is possible for the mediation percentage to be an overestimation given that it may also be reflecting confounding in our cross-sectional study [59]. Secondly, the majority of variables were self-reported, potentially introducing recall and social desirability bias into the findings. Thirdly, the measure of food insecurity used in our study has not been validated and was based on two questions and did not constitute a comprehensive food insecurity measure. Next, the survey excluded those who had limited cognitive function that was severe enough to preclude the possibility to participate in the survey. Thus, the results are not generalizable to this population. Finally, it should be noted that the present data was collected over a decade ago, and it is possible that they may not necessarily reflect the current situation.

### Conclusion

In this large sample of older adults from six LMICs, food insecurity was associated with SCC, and this relationship was mediated by sleep/energy, perceived stress, and depression. Future longitudinal studies should examine whether addressing food insecurity among older adults in LMICs can improve cognitive health.

### Supplementary Information

Below is the link to the electronic supplementary material.

Supplementary file1 (DOCX 28 kb)
