# Supplementary material for: Food insecurity and subjective cognitive complaints among adults aged ≥ 65 years from low- and middle-income countries
Source: Eur J Nutr. 2023 Aug 7;62(8):3217–26. doi: 10.1007/s00394-023-03226-5 (PMC10611875; doi:10.1007/s00394-023-03226-5)
Supplement: Supplementary file 1 — Supplementary file1 (DOCX 28 kb) [file 394_2023_3226_MOESM1_ESM.docx]

**APPENDIX**

| **Table S1** Questions used to assess sleep/energy and perceived stress | |
| --- | --- |
| **Sleep and energy** | (1) Overall in the last 30 days, how much of a problem did you have with sleeping, such as falling asleep, waking up frequently during the night or waking up too early in the morning? |
|  | (2) Overall in the last 30 days, how much of a problem did you have due to not feeling rested and refreshed during the day (e.g. feeling tired, not having energy)? |
| **Perceived stress** | (1) How often have you felt that you were unable to control the important things in your life? |
|  | (2) How often have you found that you could not cope with all the things that you had to do? |

| **Table S2** Results of the factor analysis for subjective cognitive complaints | | |
| --- | --- | --- |
| k | Eigenvalues | Proportion explained |
| 1 | 1.877848 | 0.938924 |
| 2 | 0.122152 | 0.061076 |

| **Table S3** Sample characteristics by country | | | | | | | |
| --- | --- | --- | --- | --- | --- | --- | --- |
| Characteristic |  | China | Ghana | India | Mexico | Russia | South Africa |
| Age (years) | Mean (SD) | 72.3 (11.0) | 74.1 (14.1) | 71.6 (10.0) | 74.7 (15.9) | 74.2 (10.4) | 72.8 (14.6) |
| Sex | Female | 53.4 (1.0) | 48.0 (1.4) | 48.0 (1.4) | 54.9 (2.7) | 68.2 (2.1) | 60.6 (2.0) |
| Education (years) | Mean (SD) | 4.5 (9.2) | 2.7 (8.7) | 3.0 (7.0) | 4.0 (9.0) | 9.6 (6.2) | 5.3 (10.5) |
| Wealth | Poorest | 23.2 (1.2) | 19.4 (1.1) | 20.5 (1.9) | 23.1 (2.8) | 21.1 (3.3) | 21.2 (2.5) |
|  | Poorer | 19.9 (1.0) | 20.8 (1.0) | 20.5 (1.8) | 26.5 (3.2) | 24.3 (3.6) | 18.8 (1.8) |
|  | Middle | 20.3 (0.9) | 21.1 (1.3) | 18.0 (1.3) | 16.5 (1.8) | 24.6 (3.0) | 16.4 (1.8) |
|  | Richer | 19.5 (1.0) | 19.3 (1.2) | 17.0 (1.3) | 17.0 (2.0) | 14.3 (1.9) | 19.2 (1.8) |
|  | Richest | 17.1 (1.5) | 19.4 (1.3) | 24.0 (1.8) | 16.8 (2.1) | 15.7 (2.8) | 24.3 (2.6) |
| Physical activity | High | 32.0 (1.1) | 53.5 (1.8) | 35.9 (2.0) | 25.2 (2.6) | 40.8 (3.4) | 18.2 (2.0) |
|  | Moderate | 30.3 (1.1) | 12.7 (1.0) | 25.5 (1.3) | 24.1 (4.0) | 18.4 (2.0) | 13.8 (1.4) |
|  | Low | 37.6 (1.1) | 33.8 (1.6) | 38.6 (1.7) | 50.7 (3.4) | 40.9 (3.3) | 67.9 (2.3) |
| Smoking | Never | 67.8 (0.9) | 73.5 (1.3) | 42.8 (1.9) | 60.1 (2.7) | 80.3 (1.8) | 68.5 (2.0) |
|  | Current | 23.4 (0.9) | 11.7 (1.0) | 51.0 (2.0) | 17.7 (1.9) | 9.3 (1.2) | 19.8 (1.7) |
|  | Past | 8.9 (0.6) | 14.8 (1.1) | 6.2 (0.6) | 22.2 (2.6) | 10.4 (1.3) | 11.6 (1.4) |
| Alcohol consumption | Yes | 17.1 (0.9) | 26.6 (1.4) | 5.5 (0.9) | 11.4 (1.7) | 20.7 (2.1) | 10.1 (1.1) |
| Body mass index | <18.5 | 6.3 (0.5) | 20.8 (1.3) | 46.0 (1.9) | 1.1 (0.3) | 1.7 (0.9) | 4.7 (1.3) |
| (kg/m^2^) | 18.5-24.9 | 60.1 (1.2) | 55.6 (1.5) | 44.0 (1.6) | 31.9 (3.2) | 27.1 (3.0) | 23.5 (2.0) |
|  | 25.0-29.9 | 28.3 (1.0) | 16.5 (1.0) | 7.8 (0.8) | 43.8 (2.7) | 42.4 (2.6) | 28.3 (2.3) |
|  | ≥30.0 | 5.4 (0.5) | 7.2 (0.8) | 2.2 (0.4) | 23.2 (2.3) | 28.8 (2.2) | 43.5 (2.4) |
| Diabetes | Yes | 9.5 (0.6) | 3.9 (0.5) | 7.3 (1.0) | 18.8 (2.1) | 9.0 (1.5) | 12.0 (1.8) |
| Stroke | Yes | 4.8 (0.4) | 3.8 (0.5) | 2.4 (0.4) | 7.2 (1.8) | 7.6 (1.0) | 5.1 (1.2) |
| Hypertension | Yes | 69.5 (0.9) | 60.3 (1.4) | 42.3 (1.9) | 74.2 (2.4) | 82.0 (2.1) | 81.7 (1.6) |
| Anxiety | Yes | 1.0 (0.2) | 8.1 (0.8) | 21.3 (1.6) | 5.9 (1.3) | 7.2 (1.3) | 11.0 (1.7) |
| Depression | Yes | 1.0 (0.2) | 9.0 (1.2) | 14.5 (1.2) | 6.6 (1.1) | 4.3 (0.8) | 2.3 (1.0) |
| Perceived stress^a^ | Mean (SD) | 36.1 (43.6) | 53.9 (41.3) | 47.3 (42.9) | 24.5 (51.2) | 47.8 (32.6) | 48.4 (56.3) |
| Sleep/energy^a^ | Mean (SD) | 20.9 (45.8) | 35.5 (49.0) | 40.3 (44.1) | 25.4 (58.4) | 43.3 (37.5) | 30.8 (60.0) |

Abbreviation: SD Standard deviation

Data are % (standard error) unless otherwise stated.

^a^ Perceived stress and sleep/energy were based on a scale that ranged from 0 to 100 with higher scores representing worse conditions.

**Figure S1** Prevalence of different levels of food insecurity by country
